# Effects of a multicomponent exercise program on the gross and fine motor skills of healthy, sporting inactive older adults aged 60+

**DOI:** 10.3389/fragi.2026.1819415

**Published:** 2026-07-03

**Authors:** Anneke Schumacher, Marlene Krumpolt, Lucas Sannemann, Kerstin Witte

**Affiliations:** Department of Sport Science, Otto-von-Guericke-University Magdeburg, Magdeburg, Germany

**Keywords:** coordinative abilities, full-body coordination, manual dexterity, popular sports, prevention of older adults

## Abstract

This study aimed to assess the effectiveness of a multicomponent exercise program with integrated popular sports on the gross and fine motor coordination of healthy but sporting inactive adults aged 60 and above. Coordinative abilities are a key determinant of maintaining independence in later life. From February 2022 until December 2024 a 6-month pre-post intervention study with three measurement points: at baseline (t0), after 3 months (t1), and after completion of the intervention (t2) was conducted. The intervention groups started quarterly during the intervention period. In the intervention group (IG) a total of 161 older adults (68.23 ± 4.47 years; f = 107, m = 54) participated in a twice-weekly 90-min training program integrating popular sports. Gross motor skills were assessed using the Karlsruhe Health-Oriented Coordination Test (KHCT), and fine motor skills via the Motor Performance Series (MLS). Results were compared with an inactive control group (CGinactive: n = 32) and an active reference group of senior dancers (CGactive: n = 23), which was assessed cross-sectionally at baseline only. Compared to the CGinactive, the IG demonstrated significant improvements in six of the seven gross motor tasks and in two of the four fine motor tasks (group*time interaction: p < 0.05; Relative Treatment Effect = ΔRTE ≥0.12). Post-intervention, the IG’s performance approached that of the CGactive. The findings suggest that the low-threshold, multicomponent exercise program may improve coordinative abilities in previously inactive older adults. However, results should be interpreted with caution due to the non-randomized design and variability within the sample.

## 1 Introduction

Against the backdrop of demographic change, maintaining physical and cognitive performance in old age is gaining increasing importance. Although the societal perception of aging has become more positive in recent decades, portraying older adults as more active and resilient, population-based studies continue to demonstrate a high prevalence of sports inactivity among older adults (Geyer and Eberhard, 2022; Bundesministerium für Gesundheit, 2024). Coordinative abilities - i.e., the control and regulation of movement - are essential prerequisites for mobility, independence, and quality of life. In aging research, these functions are often subsumed under the broader concept of physical function, yet the term coordination emphasizes the integration of fine motor skills, such as manual dexterity (e.g., grasping movements), and gross motor performance involving whole-body or multi-segmental movements, such as balance or spatial orientation. Both domains are vital for managing daily tasks, whether at home or in traffic (Golle et al., 2019). Coordination comprises several domains, including movement coupling, differentiation, balance control, spatial orientation, rhythmization, and the ability to adapt movements to changing environmental demands (Hottenrott et al., 2022). In this study, we define coordination as the ability to integrate sensory information, plan motor actions, and execute them in a goal-directed manner, involving the interaction of sensory input, central processing, and motor output (Hottenrott et al., 2022; Wu et al., 2020).

### 1.1 Age-related decline in motor function

With increasing age, physiological degradation processes such as the loss of synapses and proprioceptors and a decline in central nervous processing speed occur (Schott et al., 2019; Illig and Pfeffer, 2010). These impairments manifest in reduced effectiveness in the spatial-temporal coordination of muscles and body segments, and in the integration of sensory and motor information (Wu et al., 2020). Both movement speed and execution precision are negatively affected (Krug et al., 2019; Lehmann et al., 2020; Hottenrott et al., 2022), increasing the risk of falls, loss of mobility, and limitations in daily motor function - particularly among physically inactive individuals (Voelcker-Rehage, 2008; Markov et al., 2023; Illig and Pfeffer, 2010).

Coordinative abilities are closely linked to physical, cognitive, and psychological capacities (Golle et al., 2019). Their relevance in aging research is further highlighted by the concept of the Motor-Cognitive Risk Syndrome, which characterizes individuals with slow gait and subjective cognitive complaints as being at increased risk for dementia (Verghese et al., 2013). Moreover, longitudinal findings demonstrate that motor and cognitive trajectories are tightly intertwined before the onset of dementia (Montero-Odasso et al., 2015; Montero-Odasso et al., 2018). Their maintenance in old age requires training across sensory input (sensory function), information processing and storage (cognition), and motor output (motor function) (Hottenrott et al., 2022; Reuter et al., 2012). The more complex the coordination task, the stronger its correlation with cognitive performance, explained by the cognitive control of movement (Freiberger and Hagen, 2001). This is particularly evident in fine motor tasks: hand and finger movements demand higher neuronal activity than gross motor movements and draw heavily on coordination, cognition, and concentration (Lehmann et al., 2020). In bimanual tasks, both hands are controlled as a coupled system, requiring precise intra- and interhemispheric coordination for high spatial-temporal accuracy (Rudisch et al., 2023). Baseline data from the present cohort, previously analyzed with regard to age- and sex-specific differences in coordinative abilities (Schumacher et al., 2026a), revealed considerable variability within the sample. Particularly tasks requiring bimanual integration and precision under time pressure differentiated between subgroups. These findings underscore the need for structured interventions targeting both fine and gross motor coordination.

Despite age-related decline, neuroplasticity remains fundamentally intact in older age. Studies show that motor learning continues to be possible, albeit requiring more repetitions and time (Voelcker-Rehage and Willimczik, 2006; Taubert et al., 2010; Illig and Pfeffer, 2010). Training interventions can enhance motor cortical function and thus improve learning capacity (Hübner, 2020). Multicomponent movement programs, combined cognitive-motor training (dual-task training), and “exergames” - which simultaneously train various motor and cognitive subsystems (e.g., balance, reaction speed, rhythmic movement, spatial orientation) -have proven effective due to their synergistic interaction effects (Wang et al., 2022; Chan et al., 2019; Mack et al., 2022). However, many motor learning studies focus on the acquisition of specific coordination skills or the short-term performance change in gross or fine motor skills, rather than broader improvements in coordinative abilities.

### 1.2 Physical inactivity and movement opportunities in Germany

Despite the well-documented benefits of physical activity, older adults in Germany remain insufficiently active (Gut et al., 2021; Buendía-Romero et al., 2025; Huang et al., 2024). WHO data show that inactivity among older adults increased by approximately 15% between 2001 and 2016, and Germany ranks among the least active countries globally, with about 40% of adults not meeting WHO activity recommendations (Guthold et al., 2018). Barriers include high psychosocial or normative entry thresholds of existing exercise programs and the lack of sustainable concepts tailored to healthy but inactive older adults (Taylor et al., 2021; Voermans et al., 2016; RKI, 2014). While physical inactivity among older adults is well documented in Germany, similar trends are observed across Europe and globally, with a substantial proportion of older adults not meeting recommended physical activity levels. This highlights the broader relevance and transferability of intervention approaches targeting this population (Guthold et al., 2018; Clemente et al., 2021).

The present study examined one potential solution for re-engaging this population: a multicomponent, low-threshold exercise program integrating recreational sports. Recreational sports differ from conventional programs in that they involve complex, variable, and spontaneous movement patterns that require the simultaneous engagement of coordination, fine and gross motor skills, and cognitive processing (Banzer et al., 2021; Osuka et al., 2019; Witte et al., 2016). They stimulate broader neuromuscular adaptations, enhance motor learning, and promote functional transfer to daily-life activities -effects that are often less pronounced in traditional strength- or endurance-only interventions (Taylor et al., 2021; Wei et al., 2024).

Previous findings suggest that multicomponent, recreational sports-oriented approaches not only activate participants but also encourage sustained engagement: 86% of participants continued exercising after the program, with 60% incorporating recreational sports into their weekly routines (Schumacher et al., 2024; Schumacher et al., 2026b). Other popular or recreational sports like dancing, Tai Chi, karate, or Nordic walking have demonstrated diverse benefits for motor and cognitive functions in older age, particularly when compared with single-component programs (Osuka et al., 2019; Witte et al., 2016; Liu et al., 2021). However, many interventions have primarily emphasized endurance, strength, balance, or reaction, while fine motor skills have remained understudied (Taylor et al., 2021). Sports with playful, spontaneous movement patterns are considered especially suitable for enhancing coordination, reaction, and balance skills (Banzer et al., 2021; Schumacher et al., 2026b).

The primary aim of this study was to investigate whether a 6-month multicomponent, recreational sports-oriented exercise program leads to significant improvements in both gross and fine motor coordination compared to an inactive control group. A secondary, exploratory aim was to compare post-intervention performance with that of long-term physically active older adults. The comparison with the active group is exploratory in nature and not intended for causal inference. It was hypothesized that (Geyer and Eberhard, 2022) the intervention group would show significantly greater improvements over time compared to an inactive control group, and (Bundesministerium für Gesundheit, 2024) post-intervention performance would approximate that of long-term active older adults.

## 2 Methods

### 2.1 Study design

The study was conducted between February 2022 and December 2024 and followed a non-randomized, quasi-experimental pre–post intervention design and included a 24-week multicomponent exercise program specifically developed for individuals newly initiating or returning to physical activity. Participants in the intervention group (IG) completed two 90-min training sessions per week in fixed groups of 10–15 participants. New intervention groups were launched quarterly. Motor performance assessments were conducted at three standardized time points: before the intervention (t0), after 3 months (intermediate; t1), and after completion of the program at 6 months (t2).

### 2.2 Sample description

A total of 226 older adults (148 female, 78 male) were recruited for the IG through local newspaper advertisements and flyers. In accordance with the ethical requirements defined by the funding framework, all individuals who met the inclusion criteria and expressed interest in participation were granted access to the intervention. Consequently, random allocation to intervention or non-intervention conditions was not feasible. A sporting inactive control group (CGinactive) did not receive any intervention but completed the same assessments and was recruited through additional newspaper articles, flyers distributed in medical practices, and local events. Furthermore, a sporting active control group (CGactive) of older adults with ≥30 years of dance-sport experience, recruited via local sports-club flyers, was assessed once at t0 to provide solely as cross-sectional reference values for coordinative performance. The CGactive served exclusively as a cross-sectional reference to contextualize performance levels and was not included in longitudinal analyses. Due to differences in recruitment, long-term activity status, and the absence of repeated measurements, comparisons with this group are exploratory and do not permit causal inference. In recent years, dance has been systematically investigated and demonstrated to exert multifaceted beneficial effects on both motor and cognitive functioning (Liu et al., 2021; Kattenstroth et al., 2013; Rehfeld et al., 2019). All participants were considered healthy aside from age-related conditions and included if aged 60+ and not regularly engaged in sports. The sports inactivity of IG and CGinactive participants was confirmed via telephone screening and a standardized questionnaire (BSA-F (Fuchs et al., 2015)), which assesses not only physical activity during work and leisure time but also sports activity. In contrast to many studies, this study specifically focused on sports inactivity, defined as the absence of regular engagement in structured sports activities, such as participation in sports clubs or organized exercise groups. In accordance with this definition, everyday physical activities - such as housework or active commuting - were not considered. Participants were excluded only in cases of motor impairments, acute illness, or use of impairing medication (assessed by a self-designed medical history questionnaire) without medical clearance.

Due to the nature of the intervention and ethical requirements defined by the funding framework, blinding of participants and instructors was not feasible.

An *a priori* power analysis targeted the primary longitudinal hypothesis comparing the IG and CGinactive. It was conducted using G*Power (Version 3.1.9.7), based on existing studies with small-to-medium training effects (Chan et al., 2019; Teraz et al., 2022), for a mixed-design ANOVA with repeated measures (2 × 3 design: two groups over three time points; α = 0.05, 1–β = 0.95, effect size f = 0.25), yielding a required total sample size of 44 participants. For the analysis of performance changes, the final sample comprised 193 participants: 161 in the IG and 32 in the CGinactive. The active control group (CGactive) was included as a cross-sectional reference only and was not part of the original power calculation. This comparison has a *post hoc* power of approximately 23% (α = 0.05), indicating a high probability of type II error. Therefore, these results are reported as exploratory and interpreted with caution.

### 2.3 Multicomponent exercise program

The 6-month exercise program consisted of 50% varied foundational regimen targeting strength, endurance, flexibility, and coordination, and 50% technical instruction in basic elements of a range of popular sports, such as bowling, table tennis, archery, and handball (listed in Supplement). Each group participated in two weekly sessions: the first comprised a combined fitness training component, led by certified project staff, utilizing gym equipment (e.g., benches, mats) as well as small apparatus such as dumbbells, resistance bands, sticks, and medicine balls. The second weekly session consisted of an introductory class presenting a variety of health- and recreational-sport activities offered by instructors from local sports clubs in Magdeburg. The training schedule was fixed for each group and identical for all participants, who trained together as a closed cohort. The popular sports modules followed a predetermined weekly rotation, ensuring that every group experienced the same sequence of activities. No prior knowledge of sports or a specific fitness level is required, as the program aims to introduce participants to various sports through basic techniques and structured playful activities. It starts from a low threshold in terms of exercise norms and increases over time, using methods such as High Intensity Interval Training (HIIT) or game-based activities to strengthen endurance. All sessions followed a standardized training manual to ensure intervention fidelity across groups. Exercises were adapted to individual performance levels through graded task difficulty, variation of equipment, and optional intensity adjustments to ensure feasibility and safety. Participant adherence was monitored through attendance lists maintained by instructors. Participants with less than 75% attendance were excluded from the final analysis. Missed sessions were not replaced but documented, and adherence rates were considered during data interpretation. Training intensity was progressively increased over the 24-week intervention through adjustments in task complexity, coordination demands, and exercise duration. While standardized training manuals ensured consistency across groups, the multicomponent nature of the program emphasized variability rather than fixed intensity zones. Sports clubs often provide structured course formats, qualified instruction, and social integration - factors that can positively influence participation and long-term adherence. For this reason, cooperation with local sports clubs was deliberately chosen as an implementation pathway in our intervention concept. Nevertheless, the positive effects of physical activity remain fundamentally independent of the organizational setting (Schumacher et al., 2024).

### 2.4 Coordination assessments

To assess whole-body coordination, the Karlsruhe Health-Oriented Coordination Test (KHCT) was used. Fine motor skills of the hands were evaluated using the Motor Performance Series (Motorische Leistungsserie, MLS) by Schoppe, administered via the Vienna Test System.

#### 2.4.1 Karlsruhe Health-Oriented Coordination Test

The motor performance test battery assesses coordinative abilities in middle and older adulthood. This standardized test procedure evaluates skill-related development based on seven subtests targeting sensory regulation during precision tasks (Table 1): (1) figure-eight tracing, (2) one-leg stand (eyes closed), (3) ball throw at a wall, (4) ball throw with rotation, (5) ball grasping, (6) backward walking, and (7) one-leg stand (narrow beam). Tasks 1–5 are evaluated qualitatively using a trichotomous rating scale (well performed, performed, not performed), while tasks 6 and 7 are assessed quantitatively based on time (s) and number of foot contacts (n). Higher scores indicate better performance for qualitative tasks, whereas lower values reflect better performance in time- or error-based tasks. The test battery can be applied in health sports, and the overall reliability (r = 0.74) is acceptable (Tittlbach et al., 2005).

**TABLE 1:** Sensory regulation demands in the test tasks of the KHCT (modified by Tittelbach et al., 2005).

Task	Task type	Request type	Parameters
(1) figure-eight tracing *	Five figure-eight movements around two cones; vestibular system	Exteroceptive/visually guided	Trichotomous rating score Points from 0–2 0 = not performed 1 = performed 2 = well performed
(2) One-leg stand (eyes closed) *	15 s on a self-selected leg; static balance; vestibular system	Interoceptive/static	
(3) Ball throw at a wall *	Catching a ball after a 360° turn; whole body	Exteroceptive/ballistic	
(4) Ball throw with rotation *	Vertical ball throw with a 360° turn; whole body	Exteroceptive/ballistic	
(5) Ball grasping *	Five repetitions of releasing and grasping a ball between the legs in a straddle position	Interoceptive/tactile-ballistically guided bimanual; whole-body	
(6) Backward walking **	Fast backward walking over a 6-m distance using tandem steps	Interoceptive; whole-body; combines precision and time pressure	Time [s]
(7) One-leg stand (narrow beam) **	60 s one-leg stand on a narrow beam; static balance	Static; whole-body	Number of errors [n]

*qualitative trichotomous assessment using the best value from two trials, ** quantitative assessment.

#### 2.4.2 Motor Performance Series (MLS)

The Motor Performance Series by Schoppe (1974) was used to examine eye-hand coordination under conditions of time pressure and, in certain instances, precision pressure. The test includes the following four subtests with increasing demands on precision pressure and simultaneous time pressure: Tapping to assess motor speed under low precision demands in number of repetitions [n], Inserting long & short pins to evaluate motor speed in tasks requiring medium to high precision based on time [s], and Line tracing to measures fine motor performance under high precision and time pressure based on an error-free time factor [s]. Task performance reflects speed and precision, with shorter completion times or higher repetition counts indicating better performance depending on the task. The MLS demonstrates high test–retest reliability across subtests, with coefficients typically ranging between 0.80 and 0.95, and an overall stability estimate of approximately r = 0.90 (Neuwirth and Benesch, 2017).

### 2.5 Statistical data analysis

Statistical analyses were conducted using R (version 4.4.3). For the analysis of longitudinal data, participants were assigned to either the IG or the CGinactive. Due to unequal sample sizes, ordinally scaled data in the KHCT, the presence of outliers, and non-normal distribution (skewness) of the data, the Brunner-Langer model (F1-LD-F1 model; nparLD package) - a nonparametric alternative to repeated-measures mixed ANOVA - was applied (Noguchi et al., 2012). Main effects (Group, Time) and interaction effects (Group*Time) were evaluated using Wald-type statistics (WTS), ANOVA-type statistics (ATS), and a modified ANOVA-type statistic with Box approximation, all based on rank transformation (Brunner et al., 2002). In the case of significant results, *post hoc* tests for pairwise comparisons with Bonferroni correction were employed. The comparison between the post-intervention measurement (t2) of the IG and the pre-intervention measurement (t0) of the CGactive was conducted separately using the Mann-Whitney U test. A significance level of α = 0.05 was adopted. Significant effects identified via the F1-LD-F1 model were interpreted based on the ΔRTE (Relative Treatment Effect) as follows: 0.01 = no difference, ≥ 0.10 = small effect, ≥ 0.15 = medium effect, ≥ 0.25 = large effect. For interpreting results from *post hoc* tests and the Mann-Whitney U test, the correlation coefficient r was used: < 0.3 = small effect, 0.3 ≤ r < 0.5 = medium effect, ≥ 0.5 = large effect.

## 3 Results

### 3.1 Sample

**FIGURE 1:**
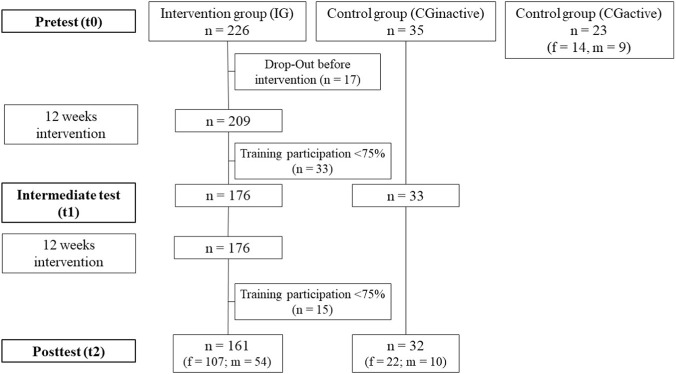
Trial-flow chart.

Of the initial 226 participants in the IG, 17 withdrew before program start, and 33 were excluded due to <75% training adherence, leaving 176 (114 female, 62 male) at t1. A further 15 dropped out before t2, resulting in valid data from 161 participants with a mean training adherence of 83.29% ± 4.78% (Figure 1). In the CGinactive, the sample size decreased from 35 to 32 participants at t2 due to personal reasons. The CGactive, consisting of older adults engaged in recreational dance sports, included a total of 23 individuals. An overview of the sample characteristics is presented in Table 2. The groups did not differ in their distribution of age or sex (χ^2^ test for sex, ANOVA for age, p > 0.05), and homogeneity of variances for the relevant metric variables was confirmed (Levene’s test, p > 0.05), indicating that the assumptions for subsequent group comparisons were met.

**TABLE 2:** Sample characteristics.

Sample	IG	CGinactive	CGactive
n	161	32	23
Age (M ± SD)	68.23 ± 4.47 years	65.63 ± 3.63 years	70.87 ± 5.21 years
Sex (%)	f: 107 (66%)	f: 22 (69%)	f: 14 (61%)
	m: 54 (34%)	m: 10 (31%)	m: 9 (39%)
BMI	28.04 ± 5.03	28.13 ± 3.85	24.78 ± 4.33
Waist-to-Height-Ratio	0.57 ± 0.08	0.59 ± 0.08	0.53 ± 0.07
Education level (ISCED 5–8)	98%	95%	97%
Years of sports inactivity (t0)	C: 27.7%	C: 21.9%	Already active
	>5: 15.8%	>5: 21.9%	
	>10: 14.9%	>10: 21.9%	
	>20: 19.8%	>20: 15.6%	
	Y: 21.8%	Y: 18.8%	
Sports activity level (before t0)	0 min per week	0 min per week	120–150 min per week

n sample size. M mean. SD, standard deviation. F female. m male. ISCED, International Standard Classification of Education C since corona-pandemic. >5, >10, >20 more than 5, 10, 20 years. Y since youth Sports activity level regularly sport sessions (BSA-F).

### 3.2 Coordinative performance outcomes

The means and standard deviations of fine and gross motor performance for the IG and CGinactive across t0–t2, as well as for the CGactive at baseline (t0), are summarized in Table 3. At baseline, the CGinactive demonstrated higher performance levels in tasks such as Ball Throw with Rotation, One-Leg Stand (Beam), and all four MLS fine motor tasks compared to the IG. However, *post hoc* test revealed no statistically significant differences in fine or gross motor performance between the IG and the CGs at this time point, except for the One-Leg Stand and Inserting Long Pins. All between-subject effects (group) between IG and CGinactive were not relevant to the primary research questions but are reported in Supplement.

**TABLE 3:** Coordinative performance outcomes at baseline (t0), intermediate (t1) and post-test (t2) for the intervention and control groups.

Task	n	Measurement point		
Group		t0 M ± SD	t1 M ± SD	t2 M ± SD
KHCT				
*figure-eight tracing* [points] ***↑***				
IG	161	0.87 ± 0.90	1.20 ± 0.90	1.26 ± 0.84
CGinactive	32	1.06 ± 0.80	0.94 ± 0.80	1.13 ± 0.87
CGactive	23	1.39 ± 0.84	-	-
*one-leg stand (eyes closed)* [points] ***↑***				
IG	161	0.22 ± 0.43	0.34 ± 0.49	0.48 ± 0.67
CGinactive	32	0.50 ± 0.76	0.47 ± 0.72	0.22 ± 0.61
CGactive	23	0.57 ± 0.73	-	-
*ball throw at a wall* [points] *↑*				
IG	161	0.43 ± 0.75	0.77 ± 0.88	0.86 ± 0.89
CGinactive	32	0.47 ± 0.80	0.63 ± 0.79	0.47 ± 0.80
CGactive	23	1.00 ± 0.67	-	-
*ball throw with rotation* [points] *↑*				
IG	161	0.55 ± 0.77	0.76 ± 0.82	0.94 ± 0.81
CGinactive	32	0.78 ± 0.79	0.81 ± 0.82	0.66 ± 0.79
CGactive	23	0.65 ± 0.71	-	-
*ball grasping* [points] *↑*				
IG	161	0.46 ± 0.73	0.76 ± 0.85	1.01 ± 0.89
CGinactive	32	0.72 ± 0.92	0.47 ± 0.80	0.66 ± 0.90
CGactive	23	0.61 ± 0.84	-	-
*backward walking* [s] *↓*				
IG	161	15.48 ± 4.68	14.08 ± 4.01	13.67 ± 3.95
CGinactive	32	16.37 ± 5.46	16.71 ± 7.24	17.08 ± 7.56
CGactive	23	11.89 ± 4.76	-	-
*one-leg stand (narrow beam)* [n] *↓*				
IG	161	3.94 ± 6.54	2.94 ± 6.28	1.99 ± 4.00
CGinactive	32	3.16 ± 4.54	3.25 ± 5.10	3.66 ± 4.53
CGactive	23	1.57 ± 2.50	-	-
MLS				
*Inserting long pins* [s] *↓*				
IG	161	41.79 ± 5.48	40.26 ± 5.18	39.85 ± 4.93
CGinactive	32	38.72 ± 4.56	38.15 ± 5.03	39.23 ± 3.99
CGactive	23	41.55 ± 5.04	-	-
*Inserting short pins* [s] *↓*				
IG	161	52.57 ± 21.77	48.98 ± 12.38	49.07 ± 12.99
CGinactive	32	48.70 ± 7.70	48.46 ± 9.11	50.23 ± 7.10
CGactive	23	53.18 ± 10.48	-	-
*Line tracing* [s] *↑*				
IG	161	0.89 ± 0.66	0.90 ± 0.69	1.01 ± 1.23
CGinactive	32	0.84 ± 0.55	0.99 ± 1.07	1.17 ± 1.26
CGactive	23	0.55 ± 0.29	-	-
*Tapping* [n] *↑*				
IG	161	183.35 ± 20.98	186.51 ± 21.15	185.96 ± 20.46
CGinactive	32	189.25 ± 15.78	189.13 ± 15.28	185.63 ± 11.68
CGactive	23	191.74 ± 17.38	-	-

**[s]** seconds. **[n]** number of repetitions**. ↑** and **↓** indicate whether a high or low test score reflects optimal performance. **M** mean. **SD**, standard deviation.

The IG showed overall improvements across all coordinative tasks, while the CGinactive generally demonstrated declines. At later measurement points, CGinactive performance in several tasks fell below that of the IG. The following analyses examine these changes in coordinative abilities for statistical significance.

### 3.3 Comparison between IG and CGinactive

The results were analyzed using the Brunner–Langer model, with the interaction effect between groups (group*time) presented in Table 4. To facilitate interpretation of this interaction effect, within-group effects were additionally examined.

**TABLE 4:** Group*Time interaction and within-group effects of coordinative abilities (IG vs. CGinactive).

Task	Group*time effects			Within-group effects			
	χ^2^ (df)	p-value	ΔRTE	Group	z	p
		(t0, t1, t2)					Effect size r
KHCT							
*figure-eight tracing* [points] ***↑***	8.54	**0.014**	0.024	IG	26.52	${<.001}^{ab}$	2.09
				CG	3.18	0.204	0.56
*one-leg stand (eyes closed)* [points] ***↑***	13.5	**<0.001**	0.121	IG	34.61	${<.001}^{b}$	2.73
				CG	4.86	0.087	0.86
*ball throw at a wall* [points] *↑*	8.32	**0.016**	0.123	IG	29.11	${<.001}^{ab}$	2.29
				CG	3.71	0.156	0.66
*ball throw with rotation* [points] *↑*	14.09	**<0.001**	0.176	IG	25.06	${<.001}^{b}$	1.98
				CG	2.38	0.305	0.42
*ball grasping* [points] *↑*	13.96	**<0.001**	0.184	IG	47.10	${<.001}^{ab}$	3.71
				CG	2.60	0.273	0.46
*backward walking* [s] *↓*	5.66	0.059	0.153	IG	27.73	${<.001}^{ab}$	2.19
				CG	1.47	0.481	0.26
*one-leg stand (narrow beam)* [n] *↓*	11.11	**0.004**	0.126	IG	33.65	${<.001}^{ab}$	2.65
				CG	0.65	**0.723**	0.11
MLS							
*Inserting long pins* [s] *↓*	5.84	0.053	0.123	IG	33.99	${<.001}^{ab}$	2.68
				CG	2.19	0.335	0.39
*Inserting short pins* [s] *↓*	9.42	**0.009**	0.154	IG	26.21	${<.001}^{ab}$	2.07
				CG	4.58	0.101	0.81
*Line tracing* [s] *↑*	1.15	0.563	0.014	IG	7.42	$.024^{b}$	0.58
				CG	1.24	0.537	0.22
*Tapping* [n] *↑*	9.27	**0.010**	0.101	IG	6.88	$.032^{b}$	0.54
				CG	5.42	0.067	0.96

**[s]** seconds. **[n]** number of repetitions. ↑ and ↓ indicate whether a high or low test score reflects optimal performance. **χ^2^** Test statistic of the WTS. **ΔRTE**, Relative Treatment Effect. **z** Test statistic of Friedman test with df = 2. **r** Pearson’s r.

Bold values indicate statistically significant differences (p < 0.05).

^a^ Within-group effects: significantly different between t0-t1 (statistically significant: p ≤ .05).

^b^ Within-group effects: significantly different between t0-t2 (statistically significant: p ≤ .05).

^c^ Within-group effects: significantly different between t1-t2 (statistically significant: p ≤ .05).

**FIGURE 2:**
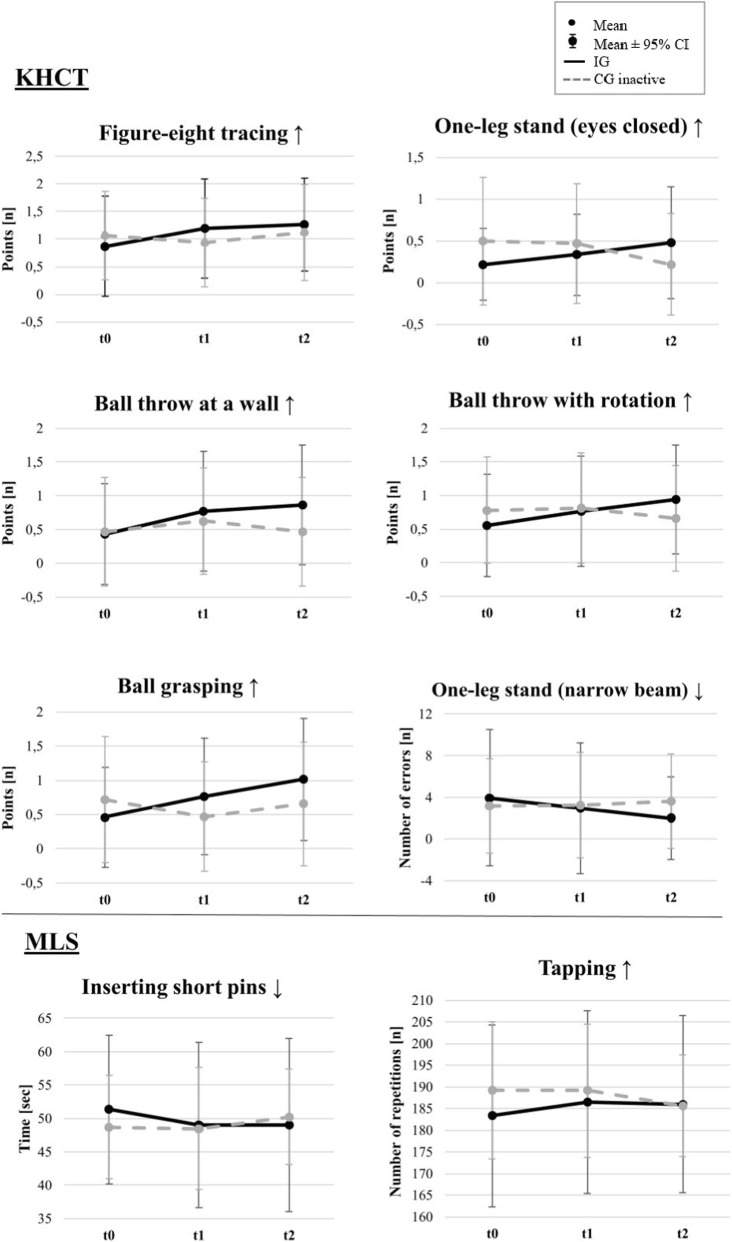
Group*time Interaction Effects for Gross Motor Coordination Outcomes in the Intervention Group (IG, n = 161) and Inactive Control Group (CGinactive, n = 32). Vertical arrows indicate whether higher (↑) or lower (↓) scores represent better performance. Significant group*time interactions were observed for all tasks shown (p < 0.05).

With regard to the gross motor tasks, all KHCT test items except backward walking showed significant interaction effects, with small to medium effect sizes (Figure 2). Post hoc analyses with Bonferroni-adjusted significance indicated that these effects were primarily driven by improvements within the IG, while the CGinactive showed no comparable changes. Improvements in the IG emerged early (t0–t1) and were maintained or further enhanced at t2, although some tasks showed delayed improvements only over the full period.

For the fine motor tasks of the MLS, significant interaction effects were found only for inserting short pins and tapping, with small to medium effect sizes (Figure 2). Within the IG, all tasks showed clear time effects, mostly with large effect sizes, whereas no significant changes were observed in the CGinactive. Improvements were particularly evident in motor speed and precision tasks, emerging either early or across the full intervention period.

### 3.4 Comparison IG und CGactive

The final performance scores of the IG at time point t2 were compared with those of the CGactive at t0 using the Mann-Whitney U test. Although the CGactive demonstrated higher baseline performance in several tasks, only a few significant differences between groups were observed. The IG achieved comparable or higher performance in multiple tasks, with significant advantages limited to selected tasks (e.g., Ball Grasping and specific fine motor precision tasks), all with small effect sizes (Table 5).

**TABLE 5:** Comparison of IG (t2) and CGactive (t0) in coordinative abilities.

Task	z	p-value	r
KHCT			
*figure-eight tracing* [points] ***↑***	0,75	0.456	0.06
*one-leg stand (eyes closed)* [points] ***↑***	0,56	0.577	0.04
*ball throw at a wall* [points] *↑*	0,89	0.374	0.07
*ball throw with rotation* [points] *↑*	−1,57	0.117	0.12
*ball grasping* [points] *↑*	−2,03	**0.043**	0.15
*backward walking* [s] *↓*	−1,87	0.061	0.14
*one-leg stand (narrow beam)* [n] *↓*	−0,12	0.903	0.01
MLS			
*Inserting long pins* [s] *↓*	1,66	0.096	0.12
*Inserting short pins* [s] *↓*	2,29	**0.022**	0.17
*Line tracing* [s] *↑*	−3,20	**0.001**	0.24
*Tapping* [n] *↑*	1,33	0.184	0.10

z z-transformed value, p-value asymptotic sig. (two-tailed), r effect size.

Bold values indicate statistically significant differences (p < 0.05).

## 4 Discussion

The present study investigated the effects of a multicomponent physical activity program on gross and fine motor functions in healthy but sporting inactive older adults. Unlike previous studies focusing on specific motor skills or short-term motor learning tasks (Roig et al., 2012; Voelcker-Rehage, 2008; Malik et al., 2024), the present intervention targeted general coordinative abilities through a broad range of recreational sport activities. Beyond coordinative improvements, previous analyses of the same intervention cohort demonstrated significant gains in selected motor skills (Schumacher et al., 2026b) and, in particular, cardiovascular fitness (Schumacher et al., 2025), suggesting that the multicomponent program exerts multidimensional benefits. The combination of cardiovascular and coordinative adaptations may be particularly relevant for maintaining functional independence in older age (Li et al., 2023).

The results indicate consistent improvements in coordinative abilities across most gross motor and selected fine motor tasks, suggesting that the multicomponent intervention effectively targets multiple domains of motor function. The intervention appears effective in stabilizing both fine and gross motor functions relevant to daily life. However, given the non-randomized study design and variability within the sample, causal conclusions regarding intervention efficacy should be interpreted cautiously. In contrast, the absence of structured physical activity appears insufficient to maintain coordinative function, highlighting the importance of regular motor stimulation in older age. Comparable effects reported for exergame- and dual-task interventions suggest that multicomponent recreational sports programs represent an effective and ecologically valid approach for improving coordinative abilities in inactive older adults, while potentially offering better transfer to activities of daily living (Pliske et al., 2016; Nayak et al., 2021; Scarmagnan et al., 2024; Li et al., 2023; Wang et al., 2022; Chan et al., 2019).

The relatively high variability observed across several outcomes likely reflects the heterogeneous motor backgrounds and functional capacities typically found in older adult populations (van der Willik et al., 2021; SINUS Markt-und Sozialforschung GmbH, 2017; Schmidt and Wrisberg, 2008). The application of nonparametric statistical methods in the present study accommodates this variability by mitigating the impact of violations of normality assumptions and heteroscedasticity.

In addition, the decline in certain tasks within the CGinactive may reflect natural age-related deterioration in motor function in the absence of structured physical activity. Motor outcomes such as gait speed, fine motor control, balance, and muscle strength show accelerated decline from later adulthood onwards when individuals do not engage in regular, targeted physical activity (van der Willik et al., 2021; Wu et al., 2020; Markov et al., 2023; Illig and Pfeffer, 2010; Teraz et al., 2022). The comparison between the IG and the CGactive is exploratory and should be interpreted cautiously because the groups differed in training history and were not assessed longitudinally. Nevertheless, the intervention group achieved comparable performance levels in several tasks. In recent years, numerous studies have demonstrated that dancing enhances both motor and cognitive skills in a particularly comprehensive manner and is considered a popular activity among older adults in Germany (Liu et al., 2021; Kattenstroth et al., 2013; SINUS Markt-und Sozialforschung GmbH, 2017; Rehfeld et al., 2019). Notably, the IG even outperformed the CGactive particularly in tasks requiring high levels of precision under time pressure. This suggests that specific elements targeting fine motor coordination, in addition to gross motor components such as those found in target-throwing sports included in the program, may be particularly effective (Schmidt and Wrisberg, 2008). Combined coordinative and physical training may improve motor performance partly through exercise-induced neuroplastic adaptations (Voelcker-Rehage et al., 2011; El-Sayes et al., 2019; Wei et al., 2024).

Regarding implementation, the sustainability of this intervention has been comprehensively evaluated in companion studies using the same cohort. After program completion participants maintained regular sports activity: most of them joined local sports clubs through membership, with preferences spanning age-typical offers and popular sports (Schumacher et al., 2024; Schumacher et al., 2026b). While formal cost-effectiveness analyses are pending, the club-based delivery leverages existing community infrastructure and sports club networks, potentially enhancing scalability for public health initiatives.

Digital technologies (like telecoaching or ehealth programs) represent complementary strategies for homebound or transportation-limited individuals but diverge from this program’s core logic of supervised, group-based recreational sports that foster social integration and structured re-entry into organized community sports (Kwon and Jang, 2024). Hybrid approaches (e.g., club training with telecoaching retention support) warrant investigation to maximize reach across diverse older adult subgroups (Subías-Perié et al., 2022; Leale et al., 2024a; Leale et al., 2024b).

### 4.1 Limitations

Strengths of the study include the large intervention sample, the use of validated assessment tools, and the practical applicability of the intervention in community settings. However, the absence of randomization, combined with the considerably smaller size of the control group, constitutes a limitation to the internal validity of the study, potentially biasing group comparisons and limiting the generalizability of the findings to the broader population. In addition, the imbalance in group sizes may have reduced the precision of between-group estimates and increased the influence of variability within the smaller control group, despite sufficient statistical power for detecting the primary longitudinal effects. Due to the ethical guidelines of the funding body, all interested individuals who met the inclusion criteria had to be granted participation in the intervention, which precluded random assignment. As a result, volunteers for the control group had to be recruited separately - without compensation - via additional newspaper advertisements. Future studies should incorporate retention tests to provide deeper insights into the long-term stability of motor learning in older adults.

### 4.2 Conclusion

The findings suggest that a multicomponent, sports-oriented exercise program may improve coordinative abilities in previously inactive older adults. However, due to the non-randomized design and sample characteristics, generalizability to other populations and settings should be interpreted with caution. The multicomponent training design may represent a feasible and scalable approach for implementation in sports clubs and community-based initiatives.

## Data Availability

The raw data supporting the conclusions of this article will be made available by the authors, without undue reservation.

## References

[B1] Banzer W. Fischer M. Groneberg D. A. (2021). Bewegung und Sport im Alter. Dtsch. Z Akupunkt. 64 (4), 272–273. 10.1007/s42212-021-00415-3

[B2] Brunner E. Domhof S. Langer F. (2002). Nonparametric Analysis of Longitudinal Data in Factorial Experiments. New York, USA: Wiley.

[B3] Buendía-Romero Á. Vetrovsky T. Hernández-Belmonte A. Izquierdo M. Courel-Ibáñez J. (2025). Residual effects of physical exercise after periods of training cessation in older adults: a systematic review with meta-analysis and meta-regression. Scand. J. Med. Sci. Sports 35 (1), e70010. 10.1111/sms.70010 39764712 PMC11705206

[B4] Bundesministerium für Gesundheit (2024). Bewegungsförderung Bei Älteren Erwachsenen in Deutschland: Bestandsaufnahme (Kurzversion).

[B5] Chan P.-T. Chang W.-C. Chiu H.-L. Kao C.-C. Liu D. Chu H. (2019). Effect of interactive cognitive-motor training on eye-hand coordination and cognitive function in older adults. BMC Geriatr. 19 (1), 27. 10.1186/s12877-019-1029-y 30691404 PMC6350349

[B6] Clemente R. Á. L. Jiménez Díaz-Benito V. Jiménez Beatty J. E. Santacruz Lozano J. A. (2021). Levels of physical activity among older adults in the european union. J. Aging Phys. Act. 29 (2), 242–249. 10.1123/japa.2020-0177 33027766

[B7] El-Sayes J. Harasym D. Turco C. V. Locke M. B. Nelson A. J. (2019). Exercise-induced neuroplasticity: a mechanistic model and prospects for promoting plasticity. Neuroscientist 25 (1), 65–85. 10.1177/1073858418771538 29683026

[B8] Freiberger E. Hagen B. (2001). Zusammenhang von Alter, kognitiven Leistungen und Bewegungskoordination. Zeitschrift für Gerontopsychologie and -psychiatrie 14 (2), 87–100. 10.1024//1011-6877.14.2.87

[B9] Fuchs R. Klaperski S. Gerber M. Seelig H. (2015). Messung der Bewegungs-und Sportaktivität mit dem BSA-Fragebogen. Z. für Gesundheitspsychologie 23 (2), 60–76. 10.1026/0943-8149/a000137

[B10] Geyer S. Eberhard S. (2022). Compression and expansion of Morbidity—Secular trends among cohorts of the same Age. Dtsch. Arztebl Int. 119 (47), 810–815. 10.3238/arztebl.m2022.0324 36300897 PMC9906028

[B11] Golle K. Mechling H. Granacher U. (2019). “Koordinative Fähigkeiten und Koordinationstraining im Sport,” in Bewegung, Training, Leistung Und Gesundheit. Editors Güllich A. Krüger M. (Berlin, Heidelberg: Springer Berlin Heidelberg), 1–24.

[B12] Gut V. Schmid J. Conzelmann A. (2021). Ein Leben lang aktiv – sportbezogene Motive und Ziele über die Lebensspanne. B and G 37 (01), 3–8.

[B13] Guthold R. Stevens G. A. Riley L. M. Bull F. C. (2018). Worldwide trends in insufficient physical activity from 2001 to 2016: a pooled analysis of 358 population-based surveys with 1·9 million participants. Lancet Glob. Health 6 (10), e1077–e1086. 10.1016/S2214-109X(18)30357-7 30193830

[B14] Hottenrott K. Hoos O. Stoll O. Blazek I. (2022). “Sportmotorische Fähigkeiten und sportliche Leistungen – Trainingswissenschaft,” in Sport. Editors Güllich A. Krüger M. (Berlin, Heidelberg: Springer Berlin Heidelberg), 563–634.

[B15] Huang K. Beckman E. M. Ng N. Dingle G. A. Han R. James K. (2024). Effectiveness of physical activity interventions on undergraduate students' mental health: systematic review and meta-analysis. Health Promot Int. 39 (3), daae054. 10.1093/heapro/daae054 38916148 PMC11196957

[B16] Hübner L. (2020). Fine Motor Performance and Motor Learning in Older Adults: Neurophysiological Processes, Effects of Acute Exercise, and Association with Physical Fitness. Chemnitz: Technische Universität Chemnitz.

[B17] Illig C. Pfeffer I. (2010). Fördert ein multidimensionales Gesundheitssportprogramm kognitive und motorische Fähigkeiten im höheren Erwachsenenalter? Sportwiss 40 (2), 110–119. 10.1007/s12662-010-0118-z

[B18] Kattenstroth J.-C. Kalisch T. Holt S. Tegenthoff M. Dinse H. R. (2013). Six months of dance intervention enhances postural, sensorimotor, and cognitive performance in elderly without affecting cardio-respiratory functions. Front. Aging Neurosci. 5, 5. 10.3389/fnagi.2013.00005 23447455 PMC3581819

[B19] Krug J. Kurth-Rosenkranz R. Voss G. Wenzel U. Witt M. (2019). Diagnostikum Der Elementaren Motorischen Schnelligkeit. Berlin: Lehmanns Media.

[B20] Kwon J. Jang J. (2024). The relationship between sports club participation, physical activity, and health behaviors among older Korean adults. Healthc. (Basel) 12 (23), 2411. 10.3390/healthcare12232411 PMC11641338 39685033

[B21] Leale I. Giustino V. Brusa J. Barcellona M. Barbagallo M. Palma A. (2024a). Effectiveness of a Sustainable Training Program combining supervised outdoor exercise with telecoaching on physical performance in elderly people. Sustainability 16 (8), 3254. 10.3390/su16083254

[B22] Leale I. Figlioli F. Giustino V. Brusa J. Barcellona M. Nocera V. (2024b). Telecoaching as a new training method for elderly people: a systematic review. Aging Clin. Exp. Res. 36 (1), 18. 10.1007/s40520-023-02648-9 38305822 PMC10837264

[B23] Lehmann W. Jüling I. (2020). “Alte Bäume bleiben beweglich – Feinmotorik im Alter,” in Auch Alte Bäume Wachsen Noch. Editors Lehmann W. Jüling I. (Berlin, Heidelberg: Springer Berlin Heidelberg), 91–108.

[B24] Li Y. Gao Y. Hu S. Chen H. Zhang M. Yang Y. (2023). Effects of multicomponent exercise on the muscle strength, muscle endurance and balance of frail older adults: a meta-analysis of randomised controlled trials. J. Clin. Nurs. 32 (9-10), 1795–1805. 10.1111/jocn.16196 34989056

[B25] Liu X. Shen P.-L. Tsai Y.-S. (2021). Dance intervention effects on physical function in healthy older adults: a systematic review and meta-analysis. Aging Clin. Exp. Res. 33 (2), 253–263. 10.1007/s40520-019-01440-y 31894561

[B26] Mack M. Stojan R. Bock O. Voelcker-Rehage C. (2022). Cognitive-motor multitasking in older adults: a randomized controlled study on the effects of individual differences on training success. BMC Geriatr. 22 (1), 581. 10.1186/s12877-022-03201-5 35840893 PMC9284902

[B27] Malik J. Główka N. Jelonek W. Stemplewski R. Maciaszek J. (2024). Effect of a juggling-based physical activity on postural stability, reaction time, and attention focus in older adults: a randomized crossover study. Eur. Rev. Aging Phys. Act. 21 (1), 15. 10.1186/s11556-024-00351-w 38822245 PMC11143604

[B28] Markov A. Hauser L. Chaabene H. (2023). Effects of concurrent strength and endurance training on measures of physical fitness in healthy middle-aged and older adults: a systematic review with meta-analysis. Sports Med. 53 (2), 437–455. 10.1007/s40279-022-01764-2 36222981 PMC9876872

[B29] Montero-Odasso M. Bherer L. Studenski S. Gopaul K. Oteng-Amoako A. Woolmore-Goodwin S. (2015). Mobility and cognition in seniors. Report from the 2008 Institute of aging (CIHR) mobility and cognition workshop. Can. Geriatr. J. 18 (3), 159–167. 10.5770/cgj.18.188 26495050 PMC4597816

[B30] Montero-Odasso M. Speechley M. Muir-Hunter S. W. Sarquis-Adamson Y. Sposato L. A. Hachinski V. (2018). Motor and cognitive trajectories before dementia: results from gait and brain Study. J. Am. Geriatr. Soc. 66 (9), 1676–1683. 10.1111/jgs.15341 29608780

[B31] Nayak A. Alhasani R. Kanitkar A. Szturm T. (2021). Dual-Task Training Program for older adults: blending gait, visuomotor and cognitive training. Front. Netw. Physiol. 1, 736232. 10.3389/fnetp.2021.736232 36925571 PMC10013153

[B32] Neuwirth W. Benesch M. (2017). Manual. Motorische Leistungsserie: Version 30 - Revision 4. Mödling, Austria.

[B33] Noguchi K. Gel Y. R. Brunner E. Konietschke F. (2012). nparLD: an R software package for the nonparametric analysis of longitudinal data in factorial experiments. J. Stat. Soft. 50 (12) 1–23. 10.18637/jss.v050.i12

[B34] Osuka Y. Kojima N. Kim M. Won C. W. Suzuki T. Kim H. (2019). Exercise type and activities of daily living disability in older women: an 8-year population-based cohort study. Scand. J. Med. Sci. Sports 29 (3), 400–406. 10.1111/sms.13336 30565317

[B35] Pliske G. Emmermacher P. Weinbeer V. Witte K. (2016). Changes in dual-task performance after 5 months of karate and fitness training for older adults to enhance fall prevention. Aging Clin. Exp. Res. 28 (6), 1179–1186. 10.1007/s40520-015-0508-z 26661888

[B36] Rehfeld K. Hökelmann A. Lehmann W. Blaser P. Knisel E. (2019). Zum Einfluss einer Tanz-und Sportintervention auf motorische und psychische Merkmale älterer Menschen. Z. für Sportpsychol. 26 (3), 130–141. 10.1026/1612-5010/a000268

[B37] Reuter E.-M. Voelcker-Rehage C. Vieluf S. Godde B. (2012). Touch perception throughout working life: effects of age and expertise. Exp. Brain Res. 216 (2), 287–297. 10.1007/s00221-011-2931-5 22080104

[B38] RKI (2014). Gesundheitliche Lage Der Männer in Deutschland. Berlin: Robert Koch-Institut.

[B39] Roig M. Skriver K. Lundbye-Jensen J. Kiens B. Nielsen J. B. (2012). A single bout of exercise improves motor memory. PLoS One 7 (9), e44594. 10.1371/journal.pone.0044594 22973462 PMC3433433

[B40] Rudisch J. Fröhlich S. Pixa N. H. Kutz D. F. Voelcker-Rehage C. (2023). Bimanual coupling is associated with left frontocentral network activity in a task-specific way. Eur. J. Neurosci. 58 (1), 2315–2338. 10.1111/ejn.16042 37165733

[B41] Scarmagnan G. S. Lino T. B. Pimentel D. E. Silva A. V. B. Da Silva Ramos I. M. Christofoletti G. (2024). Benefits of a dual-task training on motor and cognitive functions in community-dwelling older adults: a controlled clinical trial. Am. J. Phys. Med. Rehabil. 103 (5), 377–383. 10.1097/phm.0000000000002352 37903601

[B42] Schmidt R. A. Wrisberg C. A. (2008). “Motor learning and performance: a situation-based learning approach. 4,” in Champaign, Ill. Champaign, IL: Human Kinetics.

[B43] Schott N. Voelcker-Rehage C. (2019). “Motorische Entwicklung über die Lebensspanne,” in Bewegung, Training, Leistung Und Gesundheit. Editors Güllich A. Krüger M. (Berlin, Heidelberg: Springer Berlin Heidelberg), 1–32.

[B44] Schumacher A. Krumpolt M. Sannemann L. Witte K. (2024). Die Nachhaltigkeit eines Bewegungskonzepts für gesunde und körperlich inaktive Senior*innen zum Neu-oder Wiedereinstieg in den Breitensport. Präv Gesundheitsf. 20 (2), 249–255. 10.1007/s11553-024-01108-0

[B45] Schumacher A. Rahil D. Krumpolt M. Prinz A. Sannemann L. Witte K. (2025). Age- and gender-specific improvements on health-related quality of life (HRQoL) and cardiovascular fitness in healthy and sporting inactive older adults using a multidimensional exercise program. Front. Sports Act. Living 7, 1641115. 10.3389/fspor.2025.1641115 41078872 PMC12507817

[B46] Schumacher A. Krumpolt M. Prinz A. Rahil D. Sannemann L. Witte K. (2026a). Alters-und geschlechterspezifische Unterschiede der Handgeschicklichkeit bei gesunden und sportlich inaktiven älteren Erwachsenen: eine Querschnittsstudie. Z Gerontol. Geriatr. 1–6. 10.1007/s00391-025-02546-x 41518410

[B47] Schumacher A. Krumpolt M. Sannemann L. Witte K. (2026b). Influence of a sport-oriented exercise concept on motor performance and sustainability in healthy but sporting inactive older adults aged 60. Front. Sports Act. Living 8, 1702331. 10.3389/fspor.2026.1702331 41958816 PMC13057376

[B48] SINUS Markt- und Sozialforschung GmbH (2017). Die Hälfte Der Deutschen Tanzt – am Tanzfreudigsten Sind Die Jüngeren Und Die Älteren. Available online at: https://www.sinus-institut.de/media/pages/media-center/presse/die-haelfte-der-deutschen-tanzt/72ce96a380-1623079817/pressetext_welttanztag_2017_final.pdf?utm_source=chatgpt.com (Accessed June 29, 2026).

[B49] Subías-Perié J. Navarrete-Villanueva D. Gómez-Cabello A. Vicente-Rodríguez G. Casajús J. A. (2022). Health economic evaluation of exercise interventions in people over 60 years old: a systematic review. Exp. Gerontol. 161, 111713. 10.1016/j.exger.2022.111713 35104563

[B50] Taubert M. Draganski B. Anwander A. Müller K. Horstmann A. Villringer A. (2010). Dynamic properties of human brain structure: learning-related changes in cortical areas and associated fiber connections. J. Neurosci. 30 (35), 11670–11677. 10.1523/JNEUROSCI.2567-10.2010 20810887 PMC6633410

[B51] Taylor J. Walsh S. Kwok W. Pinheiro M. B. Oliveira J. S. de Hassett L. (2021). A scoping review of physical activity interventions for older adults. Int. J. Behav. Nutr. Phys. Act. 18 (1), 82. 10.1186/s12966-021-01140-9 34193157 PMC8243293

[B52] Teraz K. Šlosar L. Paravlić A. H. Bruin E. D. de Marusic U. (2022). Impact of motor-cognitive interventions on selected gait and balance outcomes in older adults: a systematic review and meta-analysis of randomized controlled trials. Front. Psychol. 13, 837710. 10.3389/fpsyg.2022.837710 35783735 PMC9245546

[B53] Tittlbach S. Kolb H. Woll A. Bös K. (2005). Karlsruher Gesundheitsorientierter Koordinationstest (KGKT). B and G 21 (06), 253–258. 10.1055/s-2005-918173

[B54] van der Willik K. D. Licher S. Vinke E. J. Knol M. J. Darweesh S. K. L. van der Geest J. N. (2021). Trajectories of cognitive and motor function between ages 45 and 90 years: a population-based Study. J. Gerontol. A Biol. Sci. Med. Sci. 76 (2), 297–306. 10.1093/gerona/glaa187 32750110 PMC7812437

[B55] Verghese J. Wang C. Lipton R. B. Holtzer R. (2013). Motoric cognitive risk syndrome and the risk of dementia. J. Gerontol. A Biol. Sci. Med. Sci. 68 (4), 412–418. 10.1093/gerona/gls191 22987797 PMC3593614

[B56] Voelcker-Rehage C. (2008). Motor-skill learning in older adults—a review of studies on age-related differences. Eur. Rev. Aging Phys. Act. 5 (1), 5–16. 10.1007/s11556-008-0030-9

[B57] Voelcker-Rehage C. Willimczik K. (2006). Motor plasticity in a juggling task in older adults-a developmental study. Age Ageing 35 (4), 422–427. 10.1093/ageing/afl025 16690635

[B58] Voelcker-Rehage C. Godde B. Staudinger U. M. (2011). Cardiovascular and coordination training differentially improve cognitive performance and neural processing in older adults. Front. Hum. Neurosci. 5, 26. 10.3389/fnhum.2011.00026 21441997 PMC3062100

[B59] Voermans S. Hombrecher M. Borgerding K. Wolters S. Ahlers G. Helbig B. (2016). Beweg Dich, Deutschland!: Tk-Bewegungsstudie 2016. Hamburg: Techniker Krankenkasse 2016.

[B60] Wang Q. Li Li Mao M. Sun W. Zhang C. Mao D. (2022). The relationships of postural stability with muscle strength and proprioception are different among older adults over and under 75 years of age. J. Exerc Sci. Fit. 20 (4), 328–334. 10.1016/j.jesf.2022.07.004 36033943 PMC9395655

[B61] Wei J.-N. Zhang M.-K. Wang Z. Liu Y. Zhang J. (2024). Table tennis experience enhances motor control in older adults: insights into sensorimotor-related cortical connectivity. Int. J. Clin. Health Psychol. 24 (2), 100464. 10.1016/j.ijchp.2024.100464 38660391 PMC11039312

[B62] Witte K. Kropf S. Darius S. Emmermacher P. Böckelmann I. (2016). Comparing the effectiveness of karate and fitness training on cognitive functioning in older adults-A randomized controlled trial. J. Sport Health Sci. 5 (4), 484–490. 10.1016/j.jshs.2015.09.006 30356535 PMC6188869

[B63] Wu R. Vito G. de Delahunt E. Ditroilo M. (2020). Age-related changes in motor function (I). Mechanical and neuromuscular factors. Int. J. Sports Med. 41 (11), 709–719. 10.1055/a-1144-3408 32365388

