# Supplementary material for: Effects of a multicomponent exercise program on the gross and fine motor skills of healthy, sporting inactive older adults aged 60+
Source: Front Aging. 2026 Jul 3;7:1819415. doi: 10.3389/fragi.2026.1819415 (PMC13376075; doi:10.3389/fragi.2026.1819415)
Supplement: Supplementary file 1 [file Table1.docx]

List of popular sports offered by local sports clubs (second session of the week):

Archery

Badminton

Basketball

Billiards

Bowling

Bosseln

Darts

Football / Soccer

Handball

Hula Fitness

Karate / Fall Prevention through Karate

Kegeln

Mini Golf

Prellball

Qi-Gong

Rowing

Tai Chi

Taiko

Dance

Table Tennis

Volleyball

Walking / Hiking

Yoga

Zumba

Table 1. Results of the comparison between the intervention (IG) and control (CG) group

|  | Group effects | | | |
| --- | --- | --- | --- | --- |
| Task | **χ² (df)** | **p -value** | **∆RTE** | **Post-Hoc** |
| **KHCT** | | | | |
| figure-eight tracing [points] **↑** | 0.34 | .559 | 0.024 | - |
| one-leg stand (eyes closed) [points] **↑** | 0.012 | .913 | 0.121 | t0: z = 3.935, p =.047, r = .28  t2: z = 6.116, p =.013, r =.44 |
| ball throw at a wall [points] ↑ | 1.87 | .171 | 0.123 | t2: z = 5.627, p =.018, r =.41 |
| ball throw with rotation [points] ↑ | 0.0013 | .971 | 0.176 | - |
| ball grasping [points] ↑ | 1.2 | .272 | 0.184 | t2: z = 4.250, p =.039, r = .31 |
| backward walking [s] ↓ | 11.23 | **< .001** | 0.153 | t1: z = 6.863, p = .009, r = .49  t2: z = 13.264, p < .001, r = .95 |
| one-leg stand (narrow beam) [n] ↓ | 0.95 | .331 | 0.126 | t2: z = 5.132, p =.023, r = .37 |
| **MLS** | | | | |
| Inserting long pins [s] ↓ | 4.59 | **.032** | 0.123 | t0: z = 7.427, p = .006, r = .53  t1: z = 4.431, p =.035, r =.32 |
| Inserting short pins [s] ↓ | 0.16 | 694 | 0.154 | - |
| Line tracing [s] ↑ | 0.085 | .770 | 0.014 | - |
| Tapping [n] ↑ | 0.6 | .439 | 0.101 | - |

**[s]** seconds. **[n]** number of repetitions. **↑** and **↓** indicate whether a high or low test score reflects optimal performance. **χ²** Test statistic of the WTS, ∆**RTE** Relative Treatment Effect, **z** Test statistic of Kruskal-Wallis test with df=1, **r** Pearson’s r
